# The forced-response method: Urgency rediscovered

**DOI:** 10.3758/s13428-025-02788-y

**Published:** 2025-08-18

**Authors:** Christian H. Poth, Anika Krause

**Affiliations:** 1 Neuro-Cognitive Psychology, Bielefeld University, PO Box 10 01 31, 33501 Bielefeld, Germany; 2 Neuro-Cognitive Psychology, and Differential Psychology, Bielefeld University, PO Box 10 01 31, 33501 Bielefeld, Germany

**Keywords:** Cognitive control, Executive function, Reaction time, Time pressure

## Abstract

Lee et al. (2025) presented their “forced-response method”, in which performance in a cognitive control task is assessed as a function of the time a target stimulus is visible (within a reaction time), in a situation with time pressure for responding. In the existing literature, this experimental approach has been referred to as the compelled-response paradigm or the urgent paradigm. Replicating earlier findings by also applying the method to the flanker task of cognitive control, Lee et al. found that urgency opens up a time window in which stimulus-driven processing overtakes cognitively controlled processing and leads to actions against current intentions (as per task instructions). Taken together, this demonstrates how the method captures the temporal evolution of performance in high resolution, providing a window into the dynamics of cognitive processing in cognitive control tasks. However, urgency may induce a specific mental state, akin to phasic alertness as a state of response and perceptual readiness. Therefore, it is an open question how the method differs from standard assessments of cognitive control and in what way it provides a better alternative.

## Introduction

Lee et al. (2025) assessed performance in a cognitive control task as a function of the time a target stimulus was visible (within a reaction time), in a situation with time pressure for responding. This allowed them to assess the temporal evolution of performance with high resolution, providing a window into the dynamics of cognitive processing. In the previous literature, this type of experimental approach was known as the *compelled-response paradigm* (e.g., Shankar et al., 2011, cf. Stanford et al., 2010) or *urgent paradigm* (for review, see Stanford & Salinas, 2021; see also Ghez et al., 1989).

In urgent tasks, participants have to respond to a target stimulus at a specific point in time. Importantly, the response is manipulated (in terms of timing relative to the target), which induces urgency (time pressure). When the target appears long before the response is required, participants have enough time to process the target and select their response accordingly. However, when the target appears only shortly before the response, participants are under time pressure for responding, they have to initiate their response before the target has been fully processed in order to respond in time (e.g., Poth, 2021; Salinas et al., 2019). By parametrically varying the time of the target onset relative to the response requirement, a *tachometric function* is obtained, showing how performance develops with increasing raw processing time (rPT), the time the target stimulus was visible within the reaction time (Salinas et al., 2019; relative to a go-signal for responding, Poth, 2021, or to trial start, Lee et al., 2025). The tachometric function shows how cognitive processing unfolds over time, enabling the study and modeling of the cognitive mechanisms that underlie processing (Salinas et al., 2019).

## Urgency in cognitive control tasks

Lee et al. (2025) replicated earlier findings by Poth (2021). Both studies combined the urgency manipulation with a flanker task (Eriksen & Eriksen, 1974), assessing cognitive control and the resolution of cognitive conflicts (for review, see Ridderinkhof et al., 2021). Participants are required to respond to a central target stimulus, an arrow pointing to the left or right, by pressing a button located corresponding to its direction. The target is flanked by distractor arrows. On congruent trials, the distractors point in the same direction as the target, on incongruent trials, the distractors point in the other direction. In this task, reaction times are typically longer on incongruent trials than on congruent trials. This congruency-effect reveals a cognitive conflict between the distractors and the target that has to be resolved before the response and leads to its delay. Both studies show that urgency changes the effects of cognitive conflicts in a qualitative fashion: Rather than delaying the correct response, incongruent compared with congruent trials open up a time window in which participants act against their intention, and respond driven by the conflicting stimuli (Lee et al., 2025; Poth, 2021). In this way, both studies provide conceptual replications of previous findings showing a similar pattern for saccadic eye movements rather than manual responses (Salinas et al., 2019). In summary, urgency manipulations now have been applied in a number of different tasks of perceptual decision-making (Shankar et al., 2011; Stanford et al., 2010) and cognitive control tasks (for review, see Stanford & Salinas, 2021), ranging from (anti-) saccade tasks (Salinas et al., 2019), to the spatial Stroop task (Poth, 2021, Krause & Poth, 2023; cf. Lu & Proctor, 1995), the numerical Stroop task (Krause & Poth, 2025a; cf. Henik & Tzelgov, 1982), the Eriksen flanker task (Poth, 2021; Krause & Poth, 2025b; cf. Eriksen & Eriksen, 1974) and the Simon task (Krause & Poth, 2025a; cf. Simon, 1969). Taken together, the findings from these tasks show that urgency opens up a time window in which responses are stimulus-driven and overpower the mechanisms for cognitive control that drive intentional action.

## Urgency to control for the speed–accuracy tradeoff in cognitive control tasks

Lee et al. (2025) offer a discussion about how urgent paradigms can solve a classic problem of assays of cognitive control based on “free” reaction times in speeded tasks: The speed–accuracy tradeoff, in which faster (lower latency) responses come at a cost of more frequent errors, and vice versa (e.g. Bogacz et al., 2009; Heitz, 2014; Wickelgren, 1977). Such tradeoffs can confound findings, for instance, when they change between experimental conditions that are compared (Liesefeld & Janczyk, 2019).

Stanford and Salinas (2021; see also Salinas et al., 2014) explain how speed and accuracy can be decoupled using the tachometric function: In contrast to overall RT, rPT captures only the portion of RT that is relevant to perceptual (and cognitive) decision-making (i.e., dependent on the presence of the target stimulus), so that the tachometric curve is unaffected by influences on motor processing that would affect RT. This discussion illustrates a general principle for improving cognitive assessments that are typically based on speeded action: The requirement to “freely” time and control the speed of response initiation in order to respond as quickly as possible is removed. As a result, the internal timing process could be removed or could become less prone to being modified by influences that would otherwise change the speed–accuracy tradeoff. For instance, the speed–accuracy tradeoff has been found to be influenced by cognitive strategies (e.g., Bogacz et al., 2010; Heitz, 2014; Recker et al., 2022) and external warning stimuli (e.g. Posner et al., 1973, assumed to increase response readiness, see below). As such, the principle of obtaining the tachometric function is similar to psychophysical approaches (Stanford & Salinas, 2021), where the temporal dynamics of perception have been assessed by physically limiting the duration of stimuli (using post-masks) while providing unlimited time for responding (e.g., Poth & Schneider, 2018, 2025; Poth et al., 2018). Complementing analysis procedures, these approaches offer experimental tools for controlling for speed–accuracy tradeoffs that can be applied to cognitive control tasks. However, speed–accuracy tradeoffs are also thought to reflect participants’ strategies of task performance (besides other aspects of information processing, e.g., information build-up, cf. Wickelgren, 1977), such as whether they emphasize speed or accuracy according to different payoff schemes (see Lee et al., 2025; for review, see Heitz, 2014). Thus, even if the speed–accuracy tradeoff was controlled for, the cognitive strategy emphasizing speed or accuracy could still impact on the cognitive processes captured by urgent tasks. For example, emphasizing the speed or accuracy in a task could affect the cognitive task set that controls task performance, and that not only affects motor processing, but also perceptual processing and decision-making, for instance by recruiting or modulating processes of attention and/or cognitive control (cf. Botvinick et al., 2001; Gratton et al., 2018). This should then affect performance in urgent tasks and the tachometric function. This may be especially relevant for manipulations extending across several trials or blocks of trials, for which participants learn the regularities of the tasks (e.g., the frequency of cognitive conflicts, as assessed by Lee et al., 2025), develop corresponding expectations, and adjust their cognitive task sets accordingly (e.g., to include task-unrelated stimuli, Dietze et al., 2024; to use the expected salience of task-related stimuli, or the salience of stimuli, Poth et al., 2014; or to form temporal expectations Vangkilde et al., 2012; Weinbach & Henik, 2013). The frequency of cognitive conflicts impacts on conflict resolution (proportion congruency/incongruency effects, e.g., Bugg & Crump, 2012). This could imply that associations between the stimuli of the task, the specific cognitive conflicts appearing in the task and the cognitive control operations required for conflict resolution could be formed through learning (e.g., Bugg & Crump, 2012). However, it is an open question whether applying expectations about the frequency of cognitive conflicts can affect the dominance of stimulus-driven processing in urgent situations or whether this dominance happens too early to allow such an influence (so that proportion congruency/incongruency effects appeared at later rPTs, but not at the earlier ones at which performance on incongruent trials dropped below chance). Vice versa, it is an interesting question for future research, if the temporary dominance of processing under urgency can be moved from stimulus-driven processing to more top-down task-driven processing (e.g., so that a highly trained cognitive conflict resolution becomes possible early on the tachometric function, so that the drop of performance under chance level is ameliorated).

## Does urgency induce a special state of mind?

Lee et al. propose the urgency-based method as an alternative to the standard assessment of cognitive control (p. 10). The method addresses the speed–accuracy tradeoff problem and provides additional information about processing dynamics (Stanford & Salinas, 2021). By averaging across rPTs, Lee et al. could recover well-known effects from the literature on cognitive control now in terms of response accuracy, such as effects of conflict frequency, distractor salience (see also Salinas et al., 2019), and intertrial congruency effects (i.e. larger congruency effects on trials after incongruent compared with congruent trials, see Poth, 2021, for a similar result). Thus, the approach seems suitable for assessing cognitive control (Lee et al., 2025). However, as discussed by Lee et al., requiring timed responses while varying the time of target onset as compared with standard tasks based on “free” reaction times could change the nature of the task. Besides such changes in strategic and cognitive settings that govern task performance, the time pressure in urgent paradigms could also change the momentary mental state of the participant (Poth, 2021). That is, a state in which the motor plans of potential responses are prepared in advance and highly active, and in which the salient visual information driving one motor plan can overpower current intentions that would drive the other prepared motor plan (Salinas et al., 2019). This state seems to include a heightened physiological arousal, as suggested by positive relationships between the level of urgency and arousal-markers from pupillometry and eye movement velocity (Poth, 2021). Such a transient increase of arousal also occurs during phasic alertness, a specific state of readiness for perception and action induced by warning stimuli, and this has led to the hypothesis that urgency indeed induces a form of phasic alertness (Poth, 2021; for discussion, see Poth, 2025). Phasic alertness has been shown to improve performance in speeded choice reaction tasks, to increase congruency effects in cognitive control tasks, sometimes to induce speed–accuracy tradeoffs, and to cause an earlier and faster visual processing for perception (for review, see Poth, 2025). Given these diverse influences of temporary states of mind as phasic alertness, it is a wide open question whether the typical state in standard speeded tasks of cognitive control matches the one of urgent task variants. In addition, it is not clear yet whether the extent to which a task requires stimulus-driven or task-/goal-driven processing in turn affects the state of phasic alertness.

Using an urgent task, Lee et al. (2025) could recover classic effects on “free” reaction times in cognitive control tasks (e.g., effects of conflict frequency, or of conflicts in the previous trial) now in terms of response accuracy, by aggregating accuracy across all rPTs. This could suggest that both “free” reaction and urgent tasks capture the same cognitive processes. However, the temporary dominance of stimulus-driven processing that is reflected in the below-chance performance (e.g., Krause & Poth, 2025b; Poth, 2021; Salinas et al., 2019) is not isolated in the aggregate across rPTs. The congruency of the previous trial has been found to affect the drop of performance below chance in previous experiments (Poth, 2021), but this has not yet been shown for the other classic effects mentioned above. Urgency (and phasic alertness) could interact with the processes underlying the classic effects on cognitive control by amplifying processing asymmetries between stimulus-driven and goal-driven processing, leading to a temporary dominance of stimulus-driven processing only in case such an asymmetry exists beforehand (Krause & Poth, 2025b).

Intuitively, one’s mental state may be different, when time elapses and urgency for responding rises while one waits for the target stimulus (in urgent tasks), as compared with a situation in which one does not have to wait and can respond right away (in non-urgent, speeded tasks). However, rather than only raising a caveat, this seems to point us to a new research direction: Future work could capitalize on this and investigate state-influences in cognitive control tasks by comparing urgent and non-urgent tasks, or by combining urgent tasks with phasic alerting.

## Data Availability

Not applicable, because no empirical research is reported and because there is no original data associated with this paper otherwise.

## Code availability

Not applicable, because there is no computer code associated with this paper.
